# Supplementary material for: Association between Polymorphism in the Janus Kinase 2 (JAK2) Gene and Selected Performance Traits in Cattle and Sheep
Source: Animals (Basel). 2023 Jul 31;13(15):2470. doi: 10.3390/ani13152470 (PMC10416845; doi:10.3390/ani13152470)
Supplement: Supplementary file 1 [file animals-13-02470-s001.zip › [supplementary file] Figure S1 - S4.pdf]

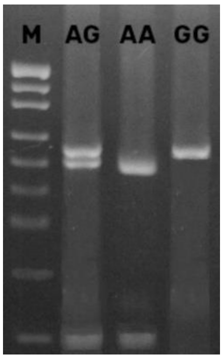

Figure S1. Electrophoretic separation of restriction fragments resulting from digestion with the *Rsa*I restriction enzyme (ACRS-PCR; bovine *JAK2*/e16/*Rsa*I polymorphism); lane 1 (M) - mass standard pUC19 DNA/*Msp*I; lane 2 - *AG* genotype; lane 3 - *AA* genotype; lane 4 - *GG* genotype.

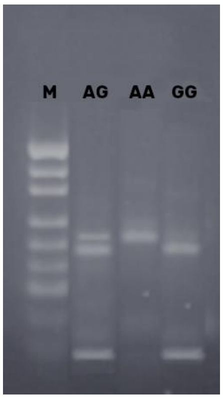

Figure S2. Electrophoretic separation of restriction fragments resulting from digestion with the *Hae*III restriction enzyme (ACRS-PCR; bovine *JAK2*/e23/*Hae*III polymorphism); lane 1 (M) - mass standard pUC19 DNA/*Msp*I; lane 2 - *AG* genotype; lane 3 - *AA* genotype; lane 4 - *GG* genotype.

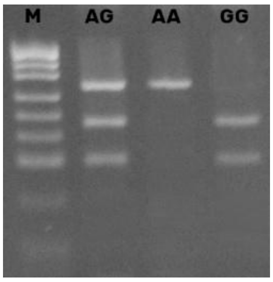

Figure S3. Electrophoretic separation of restriction fragments resulting from digestion with the *Ear*I restriction enzyme (PCR-RFLP; ovine *JAK2*/e6/*Ear*I polymorphism) polymorphic site; lane 1 (M) - mass standard pUC19 DNA/*Msp*I; lane 2 - *AG* genotype; lane 3 - *AA* genotype; lane 4 - *GG* genotype.

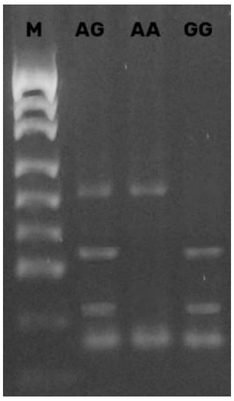

Figure S4. Electrophoretic separation of restriction fragments resulting from digestion with the *Hpy*188III restriction enzyme (PCR-RFLP; ovine *JAK2*/e24/*Hpy*188III polymorphism); lane 1 (M) - mass standard pUC19 DNA/*Msp*I; lane 2 - *AG* genotype; lane 3 - *AA* genotype; lane 4 - *GG* genotype.
