# Supplementary material for: Association between Polymorphism in the Janus Kinase 2 (JAK2) Gene and Selected Performance Traits in Cattle and Sheep
Source: Animals (Basel). 2023 Jul 31;13(15):2470. doi: 10.3390/ani13152470 (PMC10416845; doi:10.3390/ani13152470)
Supplement: Supplementary file 1 [file animals-13-02470-s001.zip › [supplementary file] Table S1.pdf]

Table S1 A summary of the *JAK2* gene fragments used for DNA sequencing and subsequent identification of polymorphic sites

Primer (5' > 3')	Tm (°C)	Product size (bp)	sequence	possible SNP	status
LEFT PRIMER<br>gaaggataacttaccagtgtcactttc<br>RIGHT PRIMER<br>aaaccaagcatatgttaataagaagaa	59.90<br>58.07	352	*Bos taurus* exon 3 (highlighted in blue)<br>1 atgctatcag tgaagacttc tttatatat**g aaggataact taccagtgtc actttc**attt<br>61 gtaactggtt tctcttacag gcaaatgttc tgaaaacgac tctgcatggg aatggcttgc<br>121 cttacaatga cagaaatgga Aggaacatcc acatcccctg tacatcagaa tggtgatatt<br>181 tctggaaatg ctaactctgt gaagcaaata gatccagtcc tacaggtcta tctTtaTcat<br>241 tcccttggga acgctgaggg ggattatctg cagtttctaa cTggagagta tgttgctgaa<br>301 gagatctgta ttgctgcttc taaagcttgt ggtaagtatt aaaaaacagt gttt**ttcttc**<br>361 **ttattaacat atgcttggtt t**attatactc taacacaatg tacctgtgta a	rs377943180<br>synonymous<br>A>G<br>p.Glu12	not detected
				rs458668038<br>synonymous<br>T>G<br>p.Leu43	not detected
				rs463147606<br>synonymous<br>T>C<br>p.Tyr44	not detected
				rs383317698<br>synonymous<br>T>G<br>p.Thr59	not detected
LEFT PRIMER<br>gggccctggacatactaagtg<br>RIGHT PRIMER<br>cctctttccaagcaaaggtt	60.75<br>58.44	265	*Bos taurus* exon 16 (highlighted in green)<br>1 **gggccctgga catactaagt g**ctcaaatat ttgtgggtta atatttgaat gtttatgcaa<br>61 ttaattttaa taggaagaaa aaacccttat tcatgggaat gtgtgCgcca aaaatattct<br>121 tcttatcaga gaagaagaca ggaagacagg aaatcctcct ttcatcaaac ttagtgatcc<br>181 tggcattagt Attacagttt tgccaaaaga cagtaagttc aacaggaatc aaatttaact<br>241 ttatt**aacct ttgcttggaa agagg**tataa aaatcatgct gttaattttt ctcaaacact	rs210330018<br>synonymous<br>C>T<br>p.Cys675	not detected
				rs210148032<br>missense<br>A>G<br>p.Ile704Val	detected and described as *JAK2*/e16/*Rsa*I
LEFT PRIMER<br>aaatcaagagttggcacatcaa<br>RIGHT PRIMER<br>tcgaaacgcttttctcatgtt	59.61<br>59.87	222	*Bos taurus* exon 23 (highlighted in dark grey)<br>1 ggta**aaatca agagttggca catcaa**gtaa ctcttttaaa tatattacag gtatgcacca<br>61 gaatcactga cagagagcaa gttttctgtg gcttcagatg tttggagctt tggagtggtt<br>121 ctGtatgaac ttttcacata tattgacaag agtaaaagcc cAccagcggt cagtatgctt<br>181 tttgtttact ttcaattttt ttttt**aacat gagaaaagcg tttcga**aaga ataatagtaa	rs461568961<br>synonymous<br>G>T<br>p.Leu1044	not detected
				rs211067160<br>synonymous<br>A>G<br>p.Pro1057	detected and described as *JAK2*/e23/*Hae*III

Primer (5' > 3')	Tm (°C)	Product size (bp)	sequence	possible SNP	status
LEFT PRIMER<br>ttgaccttgttaaatgtatatgttctg	57.85	280	*Ovis aries* exon 6 (highlighted in pink)<br>1 gag**ttgacct tgttaaatgt atatgttctg** aaaattatgc tatagattaa aatataataa<br>61 tagattaaaa tgtgatagtg aaacttaagt attttcttct atattttgtt tttactttag<br>121 tggcggcatg atttttttaca tggatggata aaagtacccg tgactcatga aacacaggaa<br>181 ga**A**tgtcttg ggatggcagt gttagatatg atgagaatag ccaaagaaaa gaatcaaac**G**<br>241 ccactggaca tctata**gctc tatcaggtaa ttttcttatg caa**atccata tgagtatgac	rs160146162<br>synonymous<br>A>G<br>p.Glu177	detected and described as *JAK2*/e6/*Ear*I
RIGHT PRIMER<br>ttgcataagaaaattacctgatagagc	60.36			rs160146160<br>synonymous<br>G>A<br>p.Thr196	detected and described as *JAK2*/e6/seq
LEFT PRIMER<br>ctcagtgtgttttgattttatgtata	54.71	253	*Ovis aries* exon 12 (highlighted in light grey)<br>1 **ctcagtgtgt tttgatttta tgtata**tttt ttcattcatt ctatcctttt tttaaacaga<br>61 caaatcaaac cttctagtct tcagaaccaa tggtgtttct **G**atgtaccaa cctcaccaac<br>121 tttacaaagg cataataa**T**g tgaaccaaat ggtgttccac aaaattagaa atgaagattt<br>181 ggtatttgta agtcaataga tgctgattat tgtctttttg tctttttaaa **caactgttct**<br>241 **tcattttcta ccc**	rs592691324<br>missense<br>G>C<br>p.Asp519His	not detected
RIGHT PRIMER<br>gggtagaaaatgaagaacagttg	57.01			rs1094682814<br>missense<br>T>G<br>p.Asn531Lys	not detected
LEFT PRIMER<br>ggacatatttcctaattcccaca	59.11	220	*Ovis aries* exon 13 (highlighted in yellow)<br>1 tt**ggacatat ttcctaattc ccaca**ttttc ttccatcctt aatcattctt tctttttttt<br>61 tttttttttt ttttggttaa agaatgaaag ccttggccaa ggaactttta caaaaatttt<br>121 taaaggtat**A** agaagagaaa taggagac**T**a tggtcagctg catgaaacag aagttctttt<br>181 aaaagttctg gataaa**gcac atagaaacta ttcagaggtt tg**tatattct ttatataatt	rs416867672<br>missense<br>A>G<br>p.Ile563Met	not detected
RIGHT PRIMER<br>caaacctctgaatagtttctatgtgc	59.66			rs1086855307<br>missense<br>T>C<br>p.Tyr570His	not detected
LEFT PRIMER<br>tctgcttgaaattaaatgtaccaaa	59.17	261	*Ovis aries* exon 24 (highlighted in red)<br>1 ttgagaat**tc tgcttgaaat taaatgtacc aaa**aatatgt cattgaaaag tgggtttgcg<br>61 tttcaggaat ttatgcgtat gattggcaat gacaaacaag gacagatgat cgtgtttcat<br>121 ttgatagaac tcct**A**aagaa taatggaaga ttac**C**gagac cagatggatg cccagatgag<br>181 gtaacaaaaa ttttttttat ccacagtaat catgcatttt ctttctcttt ttacccaagg<br>241 atttc**Aggtc agtttatgca gttcactga**a ct	rs160146116<br>synonymous<br>A>G<br>p.Leu1082	detected and described as *JAK2*/e24/*Hpy*188III
				rs1094390234<br>missense<br>C>T<br>p.Pro1089Leu	not detected
RIGHT PRIMER<br>tcagtgaactgcataaactgacc	59.30			rs429445187<br>intronic<br>A>G	detected
